# Lower incidence of new-onset severe conduction disturbances after transcatheter aortic valve implantation with bicuspid aortic valve in patients with no baseline conduction abnormality: a cross-sectional investigation in a single center in China

**DOI:** 10.3389/fcvm.2023.1176984

**Published:** 2023-06-27

**Authors:** Yuehuan Li, Ruobing Lei, Jiawei Zhou, Jiangang Wang, Haibo Zhang

**Affiliations:** ^1^ Department of Cardiac Surgery, Beijing Anzhen Hospital, Capital Medical University, Beijing, China; ^2^ Chevidence Lab Child & Adolescent Health, Department of Pediatric Research Institute, Children’s Hospital of Chongqing Medical University, Chongqing, China

**Keywords:** transcatheter aortic valve implantation (TAVI), bicuspid aortic valve (BAV), atrioventricular conduction disturbances, left bundle branch block (LBBB), high-grade atrioventricular block

## Abstract

**Background:**

With technological advancements, the incidence of most transcatheter aortic valve implantation (TAVI)-related complications, with the exception of conduction disturbances, has decreased. Bicuspid aortic valve (BAV) is also no longer considered a contraindication to TAVI; however, the effect of BAV on postoperative conduction disturbances after TAVI is unknown.

**Methods:**

We collected information on patients who met the indications for TAVI and successfully underwent TAVI at our center between January 2018 and January 2021. Patients with preoperative pacemaker implantation status or conduction disturbances (atrioventricular block, bundle branch block, and intraventricular block) were excluded. Based on imaging data, the patients were categorized into the BAV group and the tricuspid aortic valve (TAV) group. The incidence of new perioperative conduction disturbances was compared between the two groups.

**Results:**

A total of 187 patients were included in this study, 64 (34.2%) of whom had BAV. The incidence of third-degree block in the BAV group was 1.6%, which was lower than that (13.0%) in the TAV group (*P* < 0.05). Multivariate logistic regression results showed that the risk of third-degree conduction disturbances was 15-fold smaller in the BAV group than that in the TAV group [relative risk (RR) = 0.067, 95% CI = 0.008–0.596, *P* < 0.05]. The risk of other blocks in the BAV group was about half of that in the TAV group (RR = 0.498, 95% CI = 0.240–1.032); however, the difference was not statistically significant (*P* > 0.05).

**Conclusion:**

The present study found that patients with BAV had a lower rate of third-degree conduction disturbances after TAVI than patients with TAV.

## 1. Introduction

Transcatheter aortic valve implantation (TAVI) has become an accepted alternative for treating patients with severe aortic valve disease at all risk levels (1, 2). With advances in surgical techniques and prosthesis, the incidence of many complications after TAVI has decreased significantly (3, 4); however, the incidence of new-onset conduction disturbances (NOCDs) such as left bundle branch block (LBBB) and high-grade atrioventricular block (AVB) remains high (5), which could decrease left ventricular ejection fraction (LVEF) and increase the need of permanent pacemaker (PPM) implantation (6–8). The incidence of new-onset LBBB, reported as the most frequent complication after TAVI (9), ranges from 4% to 65% depending on the valve type, and the overall rate of PPM implantation with new-generation valves ranged from 2.3% to 36.1% (10).

Bicuspid aortic valve (BAV) is the most common congenital cardiac anomaly in adults. Previously, because of the anatomical challenges of TAVI, aortic stenosis patients with BAV were excluded from the indications (11). Following the update on the transcatheter heart valve (THV), TAVI is gradually being performed in patients with BAV and has shown clinical outcomes comparable to those of patients with tricuspid aortic valve (TAV). For example, Forrest et al. (12) showed no difference in the rate of mortality at 30 days and 1 year and the rate of paravalvular leak between the BAV and TAV groups (1). However, controversial findings have been reported regarding the incidence of conduction disturbances. A previous study (13) concluded that BAV stenosis increases the risk of conduction disturbances because of the short length of the membranous septum. In contrast, we observed no increased risk of conduction disturbances in patients with BAV in clinical practice in our center. Therefore, the present study aimed to assess whether there is a difference in the incidence of conduction disturbances after TAVI in aortic stenosis patients with BAV as compared to that in patients with TAV and to investigate the associated factors.

We present the following article in accordance with the STROBE reporting checklist.

## 2. Methods

### 2.1. Clinical data

This retrospective cohort study collected information on patients admitted to our center between January 2018 and January 2021 who met the indications for TAVI and successfully received TAVI (*n* = 199). Patients with preoperative PPM status (*n* = 4) or with conduction disturbances (AVB, bundle branch block, and intraventricular block) (*n* = 8) were excluded, including six cases of complete right bundle branch block (RBBB) and two cases of intraventricular block. This study was approved by the Ethics Committee of Beijing Anzhen Hospital, Capital Medical University (2022083X). Written informed consent was obtained from patients before surgery.

### 2.2. Preoperative evaluation and grouping

The conditions of all patients were discussed preoperatively by multidisciplinary teams, with a focus on indications, surgical options, and contraindications for TAVI and with full consideration of the patient’s preferences. Overall and aortic valve morphology and functional status were assessed by transthoracic echocardiography (TTE). The crucial parameters included atrial and ventricular internal diameters, ventricular wall thickness, and LVEF. Morphological parameters of the aortic valve included annular inner diameter, number of leaflets, and degree of calcified lesions. Moreover, the hemodynamic parameters included effective orifice area, peak flow velocity, and mean/maximum transvalvular pressure difference.

**Figure 1:**
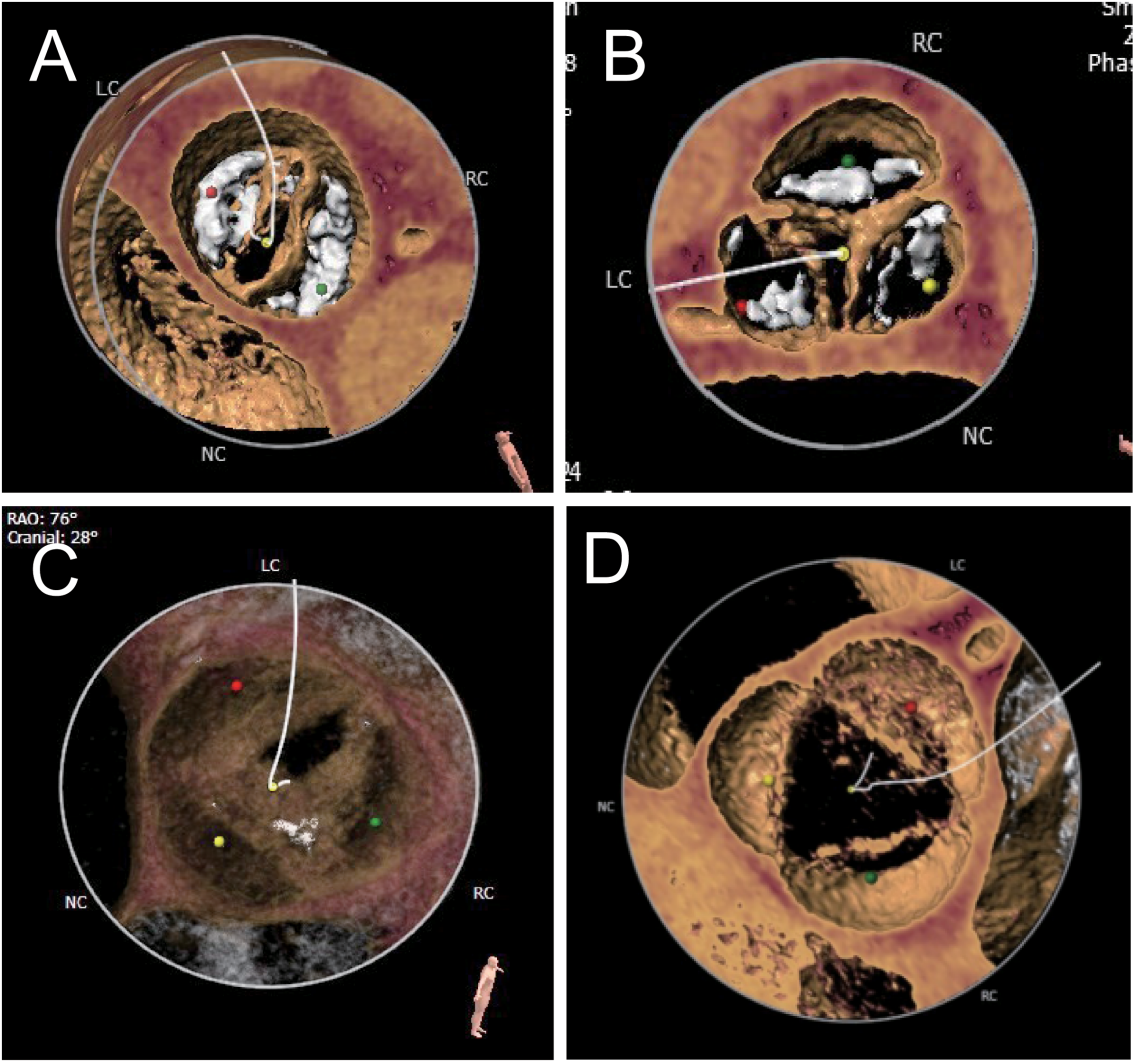
Different anatomical shapes of aortic valves. According to the Sievers classification, the bicuspid aortic valve is classified as type 0 (**A**), type I (**B**), or type II (**C**). The typical morphology is three sinuses and three leaflets (**D**).

The number of aortic valve leaflets was determined by combining CT images and ultrasound dynamic images, and the patients were then categorized into the BAV group, further categorized into type 0, type 1, and type 2 according to the Sievers classification (14), and the TAV group (Figure 1). The groups were based on the number of leaflets and sinuses determined by ultrasound and imaging physicians to avoid misclassification bias.

### 2.3. Surgical strategy

The corresponding anesthesia was selected according to the method of surgical access. For transapical approach, tracheal intubation under general anesthesia is mandatory; for the transfemoral or subclavian artery approaches, local anesthesia with monitored anesthesia care or laryngeal mask airway under general anesthesia can be chosen. A central venous catheter and temporary pacing electrodes were prepositioned in the right jugular vein. Five transcatheter valves were available during the study period: Venus-A (Qiming Medical, Hangzhou, China), J-Valve (JC Medical, Suzhou, China), TaurusElite (Peijia Medical, Suzhou, China), SAPIEN 3 (Edwards Lifesciences, Irvine, CA, United States), and VitaFlow™ system (MicroPort®, Shanghai, China). Except for SAPIEN 3, all others are self-expanding valves with nitinol stents. J-Valve and SAPIEN 3 are short THVs, while the other three are long ones. J-Valve was approved by China's National Medical Products Administration (NMPA) in 2017 for the dual indication of aortic stenosis and aortic regurgitation. Edwards received approval to launch the SAPIEN 3 valve in China on June 8, 2020. Patients with pure aortic regurgitation were implanted with the J-Valve using a transapical approach. Balloon pre-dilation is usually necessary except in lesions with pure aortic regurgitation or relatively mild stenosis. In most cases, a rapid-pacing state was required to release the stent valve. The choice of post-dilation was based on post-release valve morphology, perivalvular leak, and transvalvular flow rate.

The optimal goal of modest annulus area oversizing is usually 10%–25% for self-expandable THVs (15) and 5%–10% for balloon-expandable valves (16). Our center uses a size-reduction strategy setting the oversizing of the annular area to 5%–10% for self-expandable THVs or 0%–5% for balloon-expandable for most patients with bicuspid aortic stenosis. The bottom of THV is 0–2 mm lower than the annulus plane.

### 2.4. Postoperative antithrombotic therapy and primary observed outcomes

Patients without anticoagulation indications were administered antiplatelet therapy, and those with anticoagulation indications were administered warfarin therapy. Bedside electrocardiography (ECG) was reviewed on the same day after surgery. The electrocardiogram was reviewed before discharge to determine the presence of conduction disturbances and their type. If a third-degree AV block was present, a PPM was given. If only bundle branch conduction disturbances were present, the heart rate was normal, and the patient was not in discomfort, follow-up observation was continued, and discharge was approved.

### 2.5. Statistical methods

SPSS version 26.0 software was used for statistical analysis. Normally distributed measures were expressed as mean ± standard deviation, and comparisons between the BAV and TAV groups were made by two independent samples *t*-test and ANOVA. Median and interquartile range were used to describe the measures of skewed distribution. The Mann–Whitney *U* test or the Kruskal–Wallis test were used for comparison between groups. Count data were described using the number of cases and percentages, and comparisons between the groups were made using the chi-squared test or Fisher's exact test; the Bonferroni method was used to adjust the *P* value for two-by-two comparisons. An unordered multicategory logistic regression model was used to determine the relationship with new-onset conduction disturbances: we included all variables that differed significantly between the BAV and TAV groups, or between patients experiencing different types of new-onset conduction disturbances. The significance level was *α *= 0.05.

## 3. Results

### 3.1. Comparison of general information

**Figure 2:**
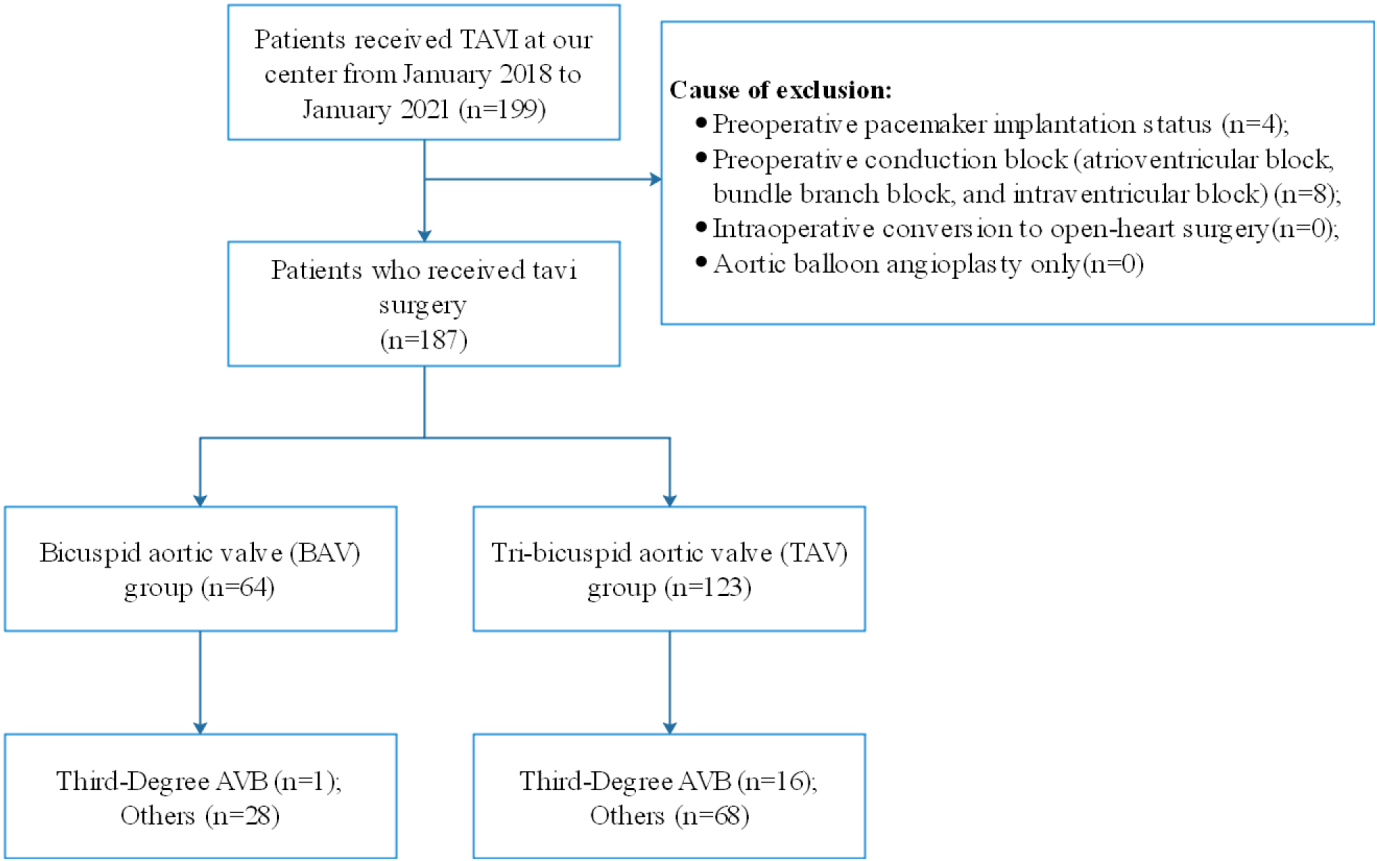
Flow diagram showing the numbers of individuals at each stage of the study. TAV, tricuspid aortic valve; BAV, bicuspid aortic valve; AVB, atrioventricular block; TAVI, transcatheter aortic valve implantation.

A total of 187 patients were included in this study, of whom 64 (34.2%) had BAV (42 patients of type 0, 20 of type 1, and two of type 2) (Figure 2). Comparison of the baseline data between the two groups revealed significant differences in hypertension, degree of regurgitation, left ventricular end-diastolic internal diameter (LVEDd), stent size, and valve type between the BAV and TAV groups (Table 1). Different types of transcatheter valves have different sizes, and the valve types involved in this study ranged from 21 to 32. Six (3.2%) patients had size 21, 37 (19.8%) patients size 23, 1 (0.5%) patient size 24, 6 (3.2%) patients size 25, 70 (37.4%) patients size 26, 12 (6.4%) patients size 27, 51 (27.3%) patients size 29, and 4 (2.1%) patients size 32.

**Table 1:** Comparison of patient characteristics between the BAV and TAV groups.

Variable	Total (*n* = 187)	TAV (*n* = 123)	BAV (*n* = 64)	*t*/*Z*/*χ*^2^	*P*
Age (years)	72.72 ± 7.68	73.32 ± 8.1	71.58 ± 6.71	1.474	0.142
Gender					
Male	111 (59.36)	70 (56.91)	41 (64.06)	0.893	0.345
Female	76 (40.64)	53 (43.09)	23 (35.94)		
Hypertension					
No	85 (45.45)	47 (38.21)	38 (59.38)	7.605	0.006
Yes	102 (54.55)	76 (61.79)	26 (40.63)		
Diabetes					
No	146 (78.07)	92 (74.80)	54 (84.38)	2.256	0.133
Yes	41 (21.93)	31 (25.20)	10 (15.63)		
CAD					
No	120 (64.17)	80 (65.04)	40 (62.50)	0.118	0.731
Yes	67 (35.83)	43 (34.96)	24 (37.50)		
NYHA classification					
Ⅰ–II	32 (17.11)	17 (13.82)	15 (23.44)	2.745	0.098
III–IV	155 (82.89)	106 (86.18)	49 (76.56)		
AR					
None	37 (19.79)	16 (13.01)	21 (32.81)	30.876	<0.001
Mild	60 (32.09)	32 (26.02)	28 (43.75)		
Moderate	39 (20.86)	27 (21.95)	12 (18.75)		
Severe	51 (27.27)	48 (39.02)	3 (4.69)		
EF	60 (51–64)	60 (54–64)	59.5 (45.5–64.5)	−0.385	0.700
LVEDd	51.88 ± 8.71	53.15 ± 8.71	49.48 ± 8.26	2.767	0.006
Type of THV^a^					
Short	48 (25.67)	40 (32.52)	8 (12.50)	8.843	0.003
Long	139 (74.33)	83 (67.48)	56 (87.50)		
Size of THV					
≤26	120 (64.17)	69 (56.10)	51 (79.69)	10.189	0.001
>26	67 (35.83)	54 (43.90)	13 (20.31)		
Paravalvular leak					
None or trace	120 (64.2)	81 (68.6)	39 (56.5)	4.087	0.124
Mild	61 (32.6)	35 (29.7)	26 (37.7)		
Moderate	6(3.2)	2(1.7)	4(5.8)		
Severe	0(0.0)	0(0.0)	0(0.0)		

CAD, coronary artery disease; AR, aortic regurgitation; EF, ejection fraction; LVEDd, left ventricular end-diastolic diameter; THV, transcatheter heart valve; TAV, tricuspid aortic valve; BAV, bicuspid aortic valve.

Results are shown as mean ± standard deviation, or frequencies (with percentages).

^a^ Five types of THVs, categorized according to the morphology into long stents (Venus-A, VitaFlow, and TaurusElite) and short stents (self-expanding J-Valve and the balloon-expanded SAPIEN 3), were used.

### 3.2. Comparison of the occurrence of new conduction disturbances

A total of 113 (60.4%) patients developed conduction disturbances after surgery, including 17 patients with third-degree AVB and 96 patients with other types of blocks (77 patients with LBBB, and 19 patients with other blocks, including complete RBBB, first-degree AVB, left anterior branch block, and intraventricular block). The incidence of third-degree AVB in the BAV group was 1.6%, which was lower than that (13.0%) in the TAV group (*P* < 0.05) (Table 2).

**Table 2:** Comparison of the occurrence of new conduction disturbances in the BAV and TAV groups.

Emerging types of conduction disturbances	Total	TAV (*n* = 123)	BAV (*n* = 64)	*t/Z/χ* ^2^	*P*
Normal	74 (39.57)	39 (31.71)	35 (54.69)^a^	12.775	0.002
Third-degree AVB	17 (9.09)	16 (13.01)	1 (1.56)^a^		
Others^b^	96 (51.34)	68 (55.28)	28 (43.75)		

TAV, tricuspid aortic valve; BAV, bicuspid aortic valve; AVB, atrioventricular block.

^a^ Compared to TAV, *P* < 0.05.

^b^ Including complete right bundle branch block, first-degree AVB, left anterior branch block, and intraventricular block.

### 3.3. Comparison of characteristics of patients with different types of new-onset conduction disturbances

Patients with new-onset conduction disturbances were slightly older (*P* = 0.03) and had hypertension more often (*P* = 0.03) than patients without conduction disturbances (Table 3).

**Table 3:** Comparison of characteristic between patients experiencing different types of new-onset conduction disturbances.

Variable	Emerging types of conduction disturbances			*t/Z/χ* ^2^	*P*
	None (*n* = 74)	Third-degree AVB (*n* = 17)	Others (*n* = 96)		
Age, years	71.04 ± 8.04	75.53 ± 7.73	73.52 ± 7.17	3.523	0.032
Gender					
Male	45 (60.81)	8 (47.06)	58 (60.42)	1.175	0.556
Female	29 (39.19)	9 (52.94)	38 (39.58)		
Hypertension					
No	39 (52.70)	3 (17.65)	43 (44.79)	6.887	0.032
Yes	35 (47.30)	14 (82.35)	53 (55.21)		
Diabetes					
No	62 (83.78)	10 (58.82)	74 (77.08)	5.145	0.076
Yes	12 (16.22)	7 (41.18)	22 (22.92)		
CAD					
No	51 (68.92)	10 (58.82)	59 (61.46)	1.244	0.537
Yes	23 (31.08)	7 (41.18)	37 (38.54)		
NYHA classification					
I–II	13 (17.57)	1 (5.88)	18 (18.75)	1.704	0.427
III–IV	61 (82.43)	16 (94.12)	78 (81.25)		
AR					
None	11 (14.86)	5 (29.41)	21 (21.88)	^a^	0.148
Mild	32 (43.24)	3 (17.65)	25 (26.04)		
Moderate	16 (21.62)	3 (17.65)	20 (20.83)		
Severe	15 (20.27)	6 (35.29)	30 (31.25)		
EF	59.5 (46–65)	57 (55–60)	60 (53–64)	1.104	0.576
LVEDd	52.04 ± 9.85	52.94 ± 8.46	51.55 ± 7.85	0.203	0.817
Type of THV**^b^**					
Short	16 (21.62)	6 (35.29)	26 (27.08)	1.561	0.458
Long	58 (78.38)	11 (64.71)	70 (72.92)		
Size of THV					
≤26	50 (67.57)	12 (70.59)	58 (60.42)	1.264	0.531
>26	24(32.43)	5(29.41)	38(39.58)		

CAD, coronary artery disease; AR, aortic regurgitation; EF, ejection fraction; LVEDd, left ventricular end-diastolic diameter; THV, transcatheter heart valve; TAV, tricuspid aortic valve; BAV, bicuspid aortic valve; AVB, atrioventricular block.

^a^ Fisher's exact test has no statistics.

^b^ Five types of THVs, categorized by morphology into long stents (Venus-A Valve, VitaFlow®, and TaurusOne®) and short stents (self-expanding J-Valve® TA and the balloon-expanded SAPIEN 3), were used.

### 3.4. Results of the multifactorial logistic regression analysis of the BAV and TAV groups and the occurrence of new-onset conduction disturbances

Age, hypertension, degree of regurgitation, LVEDd, stent length, and valve type were included in the multifactorial unordered categorical logistic regression model. The results revealed that after adjusting for all covariables, the risk of third-degree conduction disturbances was 15-fold lower in the BAV group than in the TAV group [relative risk (RR) = 0.067, 95% CI = 0.008–0.596, *P* < 0.05]. The risk of other blocks was in the BAV group was approximately half of that in the TAV group (RR = 0.498, 95% CI = 0.240–1.032); however, the difference was not statistically significant (*P* > 0.05) (Table 4).

**Table 4:** Results of the multifactor logistic regression analysis on the occurrence of new-onset conduction disturbance.

Variables and values	Third-degree AVB vs. none			Others vs. none		
	*β*	*P*	RR (95% CI)	*β*	*P*	RR (95% CI)
BAV vs. TAV	−2.703	0.015	0.067 (0.008–0.596)	−0.697	0.061	0.498 (0.240–1.032)
Age	0.088	0.053	1.092 (0.999–1.194)	0.035	0.107	1.036 (0.992–1.082)
Hypertension (yes vs. no)	1.143	0.110	3.138 (0.773–12.737)	0.155	0.638	1.168 (0.612–2.230)
AR	−0.466	0.171	0.628 (0.322–1.224)	−0.038	0.846	0.963 (0.658–1.410)
LVEDd	0.041	0.319	1.042 (0.961–1.131)	−0.015	0.507	0.985 (0.942–1.030)
Type of THV^a^ (long vs. short)	−0.696	0.346	0.499 (0.118–2.116)	−0.204	0.635	0.816 (0.352–1.889)
Size of THV (>26 vs. ≤26)	−0.524	0.462	0.592 (0.147–2.392)	0.287	0.452	1.332 (0.631–2.810)

TAV, tricuspid aortic valve; BAV, bicuspid aortic valve; AVB, atrioventricular block; AR, aortic regurgitation; LVEDd, left ventricular end-diastolic diameter; THV, transcatheter heart valve; RR, relative risk.

^a^ Five types of THVs, categorized according to morphology into long stents (Venus-A Valve, VitaFlow®, and TaurusOne®) and short stents (including self-expanding *J*-Valve® TA and the balloon-expanded SAPIEN 3), were used.

## 4. Discussion

The main finding of this study is that BAV may be a protective factor for new-onset conduction disturbances after TAVI. The incidence of third-degree disturbance was lower in the BAV group than in the TAV group. A previous study reported that the BAV membranous septum is shorter, which increases the possibility of developing postoperative LBBB (13). Another study reported results similar to those of the present study, with a much lower proportion of patients with BAV as compared to patients with TAV among those who underwent PPM implantation (17). Next, we discuss the reasons for this paradoxical phenomenon.

The overall PPM implantation rate in the present study was 9.1%, similar to those reported in previous studies (18–21). The common cardiac conduction disturbances after TAVI range from relatively benign intraventricular conduction delay to more significant LBBB and high or complete AVB requiring PPM implantation. The PPM implantation rate after TAVI has been shown to be on average higher than that of surgical aortic valve replacement (3%–7% vs. 13%–17%) (18–21). A 12%–20% reduction in PPM rates has been observed with the self-expanding Evolut R and PRO valves (Medtronic, Minneapolis, MN, United States) (22–24), whereas the SAPIEN 3 and SAPIEN 3 Ultra valves had PPM rates as low as 4.4%–6.5%, similar to earlier balloon-expandable valves (25–27). In the present study, most patients had self-expanding valves (*n* = 182) with only a small number of balloon-expanding valves (*n* = 5), which may account for the implantation rate being in the range between the two above-mentioned reports. Anatomical factors play an important role in the development of conduction disturbances after TAVI. The bundle of His and the proximal left bundle branch are closely associated with the base of the interleaf triangle between the noncoronary and right coronary artery leaflets of the aortic valve. This part of the conduction system is anatomically close to the distal landing zone of the THV in the left ventricular outflow tract, which makes it vulnerable to injury during TAVI. Importantly, the anterior–posterior relationship of the AV node to the apex of Koch's triangle and individual differences in the length and depth of the His bundle and the left proximal bundle may modulate patients’ susceptibility to conduction system injury (28). Thus, THV may cause direct or indirect injury (including direct compression, hematoma, and ischemia) to the His bundle and proximal left bundle branch, causing LBBB and high or complete AVB (28–30). This also helps explain why patients with preoperative RBBB are more likely to have postoperatively high or complete AVB, resulting in a higher rate of PPM implantation (31). Factors such as deeper THV valve implantation, larger valve size, shorter membrane septum, and location and severity of calcified masses (32–35) can also explain high PPM after TAVI based on anatomical factors. In addition, male sex, first-degree AV block, LVEDd, QRS wave widening, advanced age, diabetes mellitus, and coronary artery disease (CAD) or prior coronary artery bypass graft surgery (CABG) were reported as preoperative predictors of PPM (21, 36). In the present study, the incidence of PPM was lower in the BAV group because in our center, a smaller pre-dilated balloon with a smaller THV and a higher release position is usually selected for BAV patients, which reduces the risk of compression of the His bundle and proximal left bundle branch.

BAV is recognized as a congenital heart defect with a 0.5%–2% prevalence in the United States (37). A study from the Chinese single-center echocardiography database showed an incidence of BAV of 0.43%, comparable to previous studies in Western populations (38). However, Jilaihawi et al. (39) reported a high percentage of BAV (47.5%) in China, which is higher than that (34.2%) noted in the present study (38, 39). Moreover, the percentage of BAV in patients who underwent isolated aortic valve replacement for aortic stenosis (AS) in a study from the Western world was 41.7% in people aged 70–79 years and 27.5% in people aged 80–89 years (40). The reason for the high occurrence of BAV among the Chinese TAVI patients may be that the patients referred for TAVI in China are on average younger than in Western countries. The mean age of patients in our study was 72.7 years, whereas the mean age in European and US TAVI registries is over 80 years (38, 39).

The trapezoidal leaflet morphology (leaflet opening is significantly smaller than the annulus), which is common in patients with BAV, predisposes the valve to compression and downward migration toward the ventricle, resulting in too deep implantation. Our center uses a size-reduction strategy when selecting balloons and THVs for most patients with bicuspid aortic stenosis. Our approach has been proven safe and effective through clinical outcomes. Because of the use of retrievable devices, this strategy is likely to be implemented successfully. Several studies (36–40) have also confirmed that intraoperative success rates, postoperative all-cause mortality, and rates of stroke, severe perivalvular leak, and hemodynamics among aortic stenosis patients with BAV who received TAVI are comparable to those among aortic stenosis patients with TAV.

The probability of LBBB after TAVI is as high as 70%, with one-third of the LBBB cases being persistent (41). The incidence of new-onset LBBB after TAVI is much higher, with rates ranging from 4% to 60% reported for first-generation THV systems (34). The incidence of new-onset LBBB with first-generation mechanically expandable Lotus valves is even higher, ranging from 50% to 75% (42, 43). As noted in previous studies, 76 patients in this study had LBBB after surgery, with an incidence of 40.6%, including 24 patients (37.5%) in the BAV group and 52 patients (42.3%) in the TAV group. According to guideline recommendations for LBBB (44), continuous cardiac monitoring until discharge and no prophylactic PPM are performed if there is no progression. New-onset LBBB after TAVI is associated with poor long-term outcomes, including high mortality (45) and repeat hospitalization rates (6, 46). Thus, as TAVI is expanded to younger and lower-risk patients with less additional risk and longer expected survival, new-onset LBBB may remain an increasing concern.

Patients with preoperative conduction disturbances and pacemaker implantation were excluded from this study and new onset of conduction disturbances after TAVI was explored. In the context of increasing the success rate of TAVI and decreasing vascular complications, post-TAVI conduction disturbances are a very prominent issue, especially in the increasingly younger, low-risk TAVI patients. Combining the results of this study and the analysis of previous literature, we found that it may not be the BAV itself but the smaller valve, with the higher plane release strategy chosen for the BAV structure, which explains the low incidence of high conduction disturbances in BAV after TAVI. This implantation strategy may help reduce the incidence of conduction disturbances and disorders after TAVI.

## 5. Limitations

This study was a single-center, nonrandomized study with likely some degree of selection bias. The sample size of the study cohort was small, and propensity-matched analysis was not performed. Moreover, the study was conducted only for the perioperative period when the incidence of new conduction disturbances was highest, and long-term follow-up was not performed.

## 6. Conclusion

The present study found that patients with BAV had a lower rate of third-degree conduction disturbances after TAVI as compared to the TAV group. However, the incidence of other conduction disturbances was similar in both groups.

## Data Availability

The raw data supporting the conclusions of this article will be made available by the authors, without undue reservation.
